# 

**Published:** 1859-11

**Authors:** 


### RECEIPTS FOR THE JOURNAL, UP TO NOVEMBER 1, 1859.

| | | |
|---|---|---|
| **Ill.**—Drs. Gore & Amerman, | v. 2, '59, | $1.50 |
| " Dr. S. B. Davis, | " | $2 |
| " " John Winchester, | " | 2 |
| " " J. S. Pashley, | " | 2 |
| " " D. W. Young, | " | 2 |
| " " James Robie, | " | 2 |
| " " G. P. Rawson, | " | 2 |
| " " A. S. Mead, balance | " | 1 |
| " " D. D. Thompson, | " | 2 |
| " " J. S. Lawrence, balance | " | 1 |
| " " N. Holton, | " | 2 |
| " " T. Seely, | " | 2 |
| " " D. J. Lee, | v. 2 & ½ 3, '59–'60, | 2 |
| " " L D. Personett, | v. 2, '59, | 2 |
| " " R. P. Henry, | v. 3, '60, | 2 |
| " " Josiah Slack, | v. 2, '[illegible]9, | 2 |
| " " J. B. Glass, | v. 1 & 2, '58–'59, | 3 |
| " " S. Long, | v. 3, '60, | 2 |
| " " E. Smith, | v. 3 & 4, '60–'61, | 4 |
| " " R. A. Perkins, | v. 2 & 3, '59–'60, | 4 |
| " " H. McFarland, | v. 2, '59, | 2 |
| " " Wm. B. Hart, | " | 2 |
| " " Dr. J. G. Tilden, | " | 2 |
| " " George Richardson, | " | 2 |
| " " J. H. Wilkinson, | " | 2 |
| " " Megler. | " | 2 |
| **nd.**—Dr. D. Rea, | " | 2 |
| " " A. J. Goldsbury, | " | 2 |
| **Ind.**—Dr. J. B. Buchtel, | v. 2, '59, | $2 |
| " " C. Palmeton, | " | 2 |
| " " Samuel Ross, | " | 2 |
| " " S. B. Kyler, | v. 2 & ½ 3, '59–'60, | 3 |
| " " C. J. Keegan, | v. 2, '59, | 2 |
| " " S. W. Puriance, | v. 1, '58, | 2 |
| " " T. A. Graham, | v. 2, '59, | 2 |
| " " M. Stahl, balance | " | 1 |
| **Wis.**—Dr. Jas. Prentice, | v. 1, 2 & ½ 3, '58–'59–'60, | 5 |
| " " J. B. Dousman, | v. 2, '59, | 2 |
| " " C. D. Coleman, | " | 2 |
| **Iowa.**—Dr. J. Muncey, | " | 2 |
| " " D. Kittle, | " | 2 |
| " " W. E. Peters, | " | 2 |
| " " H. Carpenter, | " | 2 |
| " " J. S. Green, | v. 2 & 3, '59–'60, | 4 |
| **Ohio.**—Dr. Andrew Foster, | v. 2, '59, | 2 |
| **Mo.**—Dr. R. S. Kellam, | " | 2 |
| **Penn.**—Dr. J. K. Finley, | v. 1, 2 & ½ 3. '58–'59–'60, | 5 |
| **Mich.**—Dr. Amos Crosby, | v 2. '59, | 2 |
| " " J S. Skinner, | v. 1–½ 2, '58–'59, | 3 |
| **Ark.**—Dr. S. L. Urmston, | v. 2, '59, | 2 |

**Nebraska Territory.**

| | | |
|---|---|---|
| Dr. Lorenzo Bentz, | v. 2, '59, | 2 |
| " S. J. Dunn, | " | 1 |

The Surgical Clinic of the College will be held on Saturday afternoon of each week at the College.

